# Balancing selection is common in the extended MHC region but most alleles with opposite risk profile for autoimmune diseases are neutrally evolving

**DOI:** 10.1186/1471-2148-11-171

**Published:** 2011-06-17

**Authors:** Rachele Cagliani, Stefania Riva, Uberto Pozzoli, Matteo Fumagalli, Giacomo P Comi, Nereo Bresolin, Mario Clerici, Manuela Sironi

**Affiliations:** 1 Scientific Institute IRCCS E. Medea, 23842 Bosisio Parini (LC), Italy; 2 Bioengineering Department, Politecnico di Milano, 20133 Milan, Italy; 3 Dino Ferrari Centre, Department of Neurological Sciences, University of Milan, IRCCS Ospedale Maggiore Policlinico, Mangiagalli and Regina Elena Foundation, 20100 Milan, Italy; 4 Chair of Immunology, Department of Biomedical Sciences and Technologies LITA Segrate, University of Milano, 20090 Milano, Italy; 5 Fondazione Don C. Gnocchi, IRCCS, 20148 Milano, Italy

**Keywords:** autoimmune disease, balancing selection, opposite risk profile, extended MHC region

## Abstract

**Background:**

Several susceptibility genetic variants for autoimmune diseases have been identified. A subset of these polymorphisms displays an opposite risk profile in different autoimmune conditions. This observation open interesting questions on the evolutionary forces shaping the frequency of these alleles in human populations.

We aimed at testing the hypothesis whereby balancing selection has shaped the frequency of opposite risk alleles.

**Results:**

Since balancing selection signatures are expected to extend over short genomic portions, we focused our analyses on 11 regions carrying putative functional polymorphisms that may represent the disease variants (and the selection targets). No exceptional nucleotide diversity was observed for *ZSCAN23*, *HLA-DMB*, *VARS2*, *PTPN22*, *BAT3*, *C6orf47*, and *IL10*; summary statistics were consistent with evolutionary neutrality for these gene regions. Conversely, *CDSN/PSORS1C1*, *TRIM10/TRIM40*, *BTNL2*, and *TAP2 *showed extremely high nucleotide diversity and most tests rejected neutrality, suggesting the action of balancing selection. For *TAP2 *and *BTNL2 *these signatures are not secondary to linkage disequilibrium with HLA class II genes. Nonetheless, with the exception of variants in *TRIM40 *and *CDSN*, our data suggest that opposite risk SNPs are not selection targets but rather have accumulated as neutral variants.

**Conclusion:**

Data herein indicate that balancing selection is common within the extended MHC region and involves several non-HLA loci. Yet, the evolutionary history of most SNPs with an opposite effect for autoimmune diseases is consistent with evolutionary neutrality. We suggest that variants with an opposite effect on autoimmune diseases should not be considered a distinct class of disease alleles from the evolutionary perspective and, in a few cases, the opposite effect on distinct diseases may derive from complex haplotype structures in regions with high genetic diversity.

## Background

**Figure 1:**
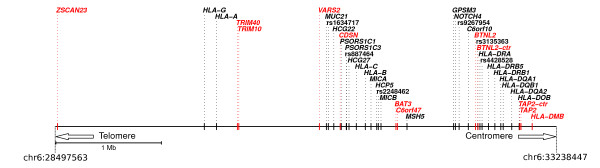
**Schematic representation of a portion of the xMHC region**. The location of all genes mentioned in the text is shown. Opposite risk SNPs are indicated with their ID if they are intergenic, otherwise the gene is indicated. The genes/gene regions we analysed are reported in red. Genomic coordinates for the region shown are chr6: 28497563-33238447 (NCBI Build 36.1).

Genome-wide association studies (GWAS) have proved powerful in unravelling the genetic component of several common diseases and complex traits, although increasing evidences [1] suggest that rare variants, which are typically not analysed in GWASs, also contribute a considerable proportion of disease risk. Through GWASs and meta-analyses, a large number of single nucleotide polymorphisms (SNPs) have been associated with distinct autoimmune conditions including Crohn's disease (CD), ulcerative colitis (UC), multiple sclerosis (MS), type 1 diabetes (T1D), rheumatoid arthritis (RA), autoimmune thyroid disease (ATD), and ankylosing spondylitis (AS). A general concept emerging from these studies is that a portion of susceptibility alleles is shared among two or more diseases, suggesting that common molecular mechanisms and pathways are involved. This may come as no surprise given the observation whereby clustering of distinct autoimmune diseases occurs within families (reviewed in [2]). However, recent evidences have also indicated that a subset of alleles displays an opposite risk profile in different autoimmune conditions with one allele predisposing to one disease while being protective for another [3,4]. The first described example concerns a nonsynonymous variant (R602W, rs2476601) in *PTPN22 *(a tyrosine phosphatase expressed in T cells): the 602W allele protects from CD but predisposes to RA, SLE (systemic lupus erythematosus), T1D (reviewed in [5]), and vitiligo [6]. Similar observations have recently been extended to several SNPs [3,4], mostly located within the extended major histocompatibility complex (xMHC) region (Figure 1).

Besides opening interesting questions as to how immune balances are maintained and modulated, these data stimulate speculations on the evolutionary forces and selective pressures shaping the frequency of these alleles in human populations.

In general, variants associated with complex traits contribute little to the overall disease risk and are therefore thought to be subjected to mild purifying selection [7]. Yet, a portion of risk alleles may be regarded as deleterious, albeit mildly, from a medical standpoint but evolutionary neutral or even beneficial. Evolutionary studies of the MHC region have mainly focused on HLA class I and II genes, that are known to be characterized by extreme polymorphism levels maintained by natural selection (reviewed in [8]). Conversely, the evolutionary history of non-HLA genes has rarely been investigated.

Here we aimed at testing the hypothesis [4] whereby alleles with opposite risk profiles for autoimmune diseases have been maintained by balancing selection, possibly due to antagonistic pleiotropy, and to describe the evolutionary pattern of several non-HLA genes located in the human xMHC. Our data indicate that long-standing balancing selection has characterized the evolutionary history of non-HLA genes located in the xMHC but only a minority of alleles with opposite risk profile can be regarded as targets of natural selection in human populations.

## Results

### Identification of alleles with opposite risk profile and selection of candidate genomic regions

**Figure 2:**
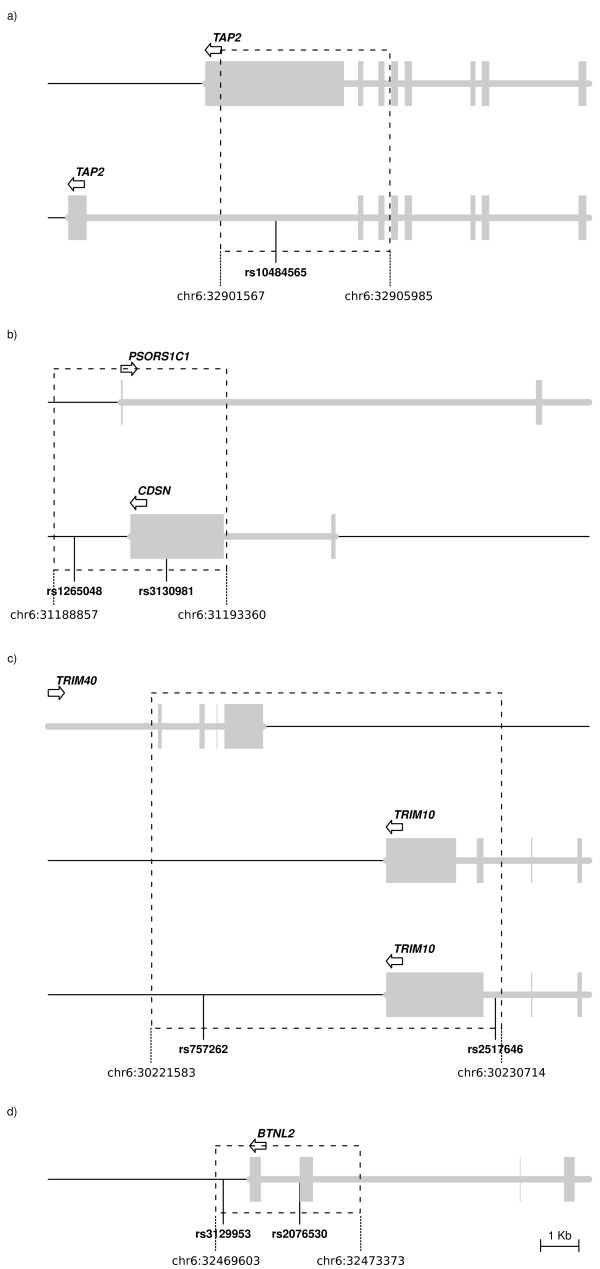
**Schematic representation of the gene regions we resequenced in *TAP2*, *TRIM10/TRIM40*, *CDSN/PSORS1C1 *and *BTNL2***. Transcribed regions are shown in grey; different transcripts either from the same or from different genes are shown separately. The direction of transcription is indicated by the arrows. The location of SNPs with opposite risk profile is reported.

Two recent studies [3,4] have identified several alleles with opposite risk profiles for autoimmune diseases (Table 1). In order to search for additional instances, we analysed published GWAS http://www.genome.gov/26525384 and, in addition to the aforementioned variant in *PTPN22*, we identified rs744166 in *STAT3 *and rs2201841 in *IL23R *(Table 1). The aim of our study is analysing the selective processes acting on variants with an opposite effect on two or more diseases and testing the hypothesis whereby balancing selection has shaped the frequency of a portion of these alleles. Balancing selection is a process that maintains genetic variability in human populations and its signatures, due to recombination and mutation, are expected to extend over relatively short genomic regions (reviewed in [9]). Since variants identified in association studies often represent genetic markers rather than causal polymorphisms, we analysed the SNPs reported in table 1 and their surrounding genomic regions for the presence of putative functional polymorphisms that may represent the causal variant and, possibly, the selection target. Details on functional annotation are reported in table 1 as well as in Figure 2 (and in additional file 1). Variants located in intergenic or relatively large intronic regions were not analysed due to the difficult inference of functional significance. Also, the polymorphism located in *MICA *was excluded as nucleotide diversity at this locus has been extensively investigated, although a clear picture of the selective or non-selective forces responsible for the presence of multiple alleles is still missing [10]. In the case of *TAP2*, the analysed region was extended so as to include a region that undergoes haplotype-specific alternative splicing [11]. SNPs located in close physical proximity were analysed as a single region: this was the case for rs3130981 and rs1265048 (in *CDSN/PSORS1C1)*, rs2076530 and rs3129953 *(*in *BTNL2)*, and rs757262 and rs2517646 *(*in *TRIM10/TRIM40)*.

**Table 1:** SNPs with opposite risk profiles in different autoimmune diseases

SNP	Gene	Chr^a^	A1^b^	Annotation	Diseases^c^	Ref
					RA-AS-	
rs11752919	*ZSCAN23*	*6*	C	in small intron		[3]
					T1D/ATD-MS	
				Asp527Asn (in *CDSN*); less than 2.6	RA-AS-	
rs3130981	*CDSN/PSORS1C1*	*6*	A			[3]
				kb from rs1265048	T1D/ATD-MS	
				intergenic; 1.2kb upstream of	RA-AS-	
rs1265048		*6*	C			[3]
				*PSORS1C1*	T1D/ATD-MS	
					RA-AS-	
rs151719	*HLA-DMB*	*6*	G	in small intron		[3]
					T1D/ATD-MS	
					ATD-MS/RA-	
rs10484565	*TAP2*	*6*	T	in 3' UTR		[3]
					AS-T1D	
					RA-AS-	
0rs1264303	*VARS2*	*6*	G	in 5'UTR		[3]
					T1D/ATD-MS	
					RA-AS-	
rs2076530	*BTNL2*	*6*	G	splice site altering/protein truncation		[3]
					T1D/ATD-MS	
				intergenic; 0.7 kb downstream	RA-AS-MS/T1D-	
rs3129953		*6*	T			[3]
				*BTNL2*	ATD	
					RA-AS-	
rs757262	*TRIM40*	*6*	T	Thr183Met		[3]
					T1D/ATD-MS	
				intronic; close to alternatively spliced	RA-AS-T1D-	
rs2517646	*TRIM10*	*6*	G			[3]
				exon	ATD/MS	
					RA-AS-	
rs2071286	*NOTCH4*	*6*	A	intronic		[3]
					T1D/ATD-MS	
					CD/T1D-RA-	
rs2476601	*PTPN22*	*1*	T	Arg620TRP		[6,60-64]
					SLE-GD	
				about 1kb downstream the		
rs3024505	*IL10*	*1*	A		T1D/CD-UC	[4]
				transcription end site		
				about 1kb downstream the		
rs917997	*IL18RAP*	*2*	T		T1D/CD	[4]
				transcription end site		
rs9388489		*6*	G	intergenic	CD/T1D	[4]
rs4788084		*16*	T	intergenic	T1D/CD	[4]
					T1D-ATD-	
rs1063635	*MICA*	*6*	A	Gln274Arg		[3]
					MS/AS	
rs1634717		*6*	A	intergenic	RA/T1D-ATD-	[3]
					MS	
					RA-AS-MS/T1D-	
rs204991	*GPSM3*	*6*	C	intronic		[3]
					ATD	
rs2242655	*C6orf47*	*6*	C	Lys92Asn	ATD-MS/RA-	[3]
					AS-T1D	
					ATD-MS/RA-	
rs2299851	*MSH5*	*6*	T	Intronic		[3]
					AS-T1D	
					AS/RA-T1D-	
rs2248462		*6*	A	intergenic		[3]
					ATD-MS	
				intronic; close to alternatively spliced	RA-AS-	
rs2844463	*BAT3*	*6*	T			[3]
				exon	ATD/T1D-MS	
					RA-AS-MS/T1D-	
rs3135363		*6*	C	intergenic		[3]
					ATD	
					RA-AS-MS/T1D-	
rs4428528		*6*	C	intergenic		[3]
					ATD	
					MS/RA-AS-T1D-	
rs887464		*6*	A	intergenic (upstream PSORS1C3)		[3]
					ATD	
					ATD-MS/RA-	
rs9267954		*6*	T	intergenic		[3]
					AS-T1D	
rs2201841	*IL23R*	*1*	G	intronic	UC/psoriasis	[65,66]
rs744166	*STAT3*	*17*	G	intronic	CD/MS	[67,68]

^a ^chromosome

^b ^minor allele in CEU

^c ^diseases are shown in order depending on the predisposing/protective effect of the minor allele.

As for rs917997, located downstream *IL18RAP*, the SNP was not considered as the gene has previously been shown to be subjected to balancing selection [12]. Finally, rs3024505 (downstream *IL10*) lies in a region resequenced by the SeattleSNPs program and data were therefore retrieved from their website. A signature of balancing selection at the promoter region of IL10 had previously been described [13]. Resequencing data for *STAT3 *are also publicly available (from the SeattleSNPs program website) but the opposite-risk SNP was not analysed as it is located within a resequencing gap in the long intron 1.

### Nucleotide diversity and neutrality tests

As reported in table 2, at least 2 kb encompassing each selected SNP(s) (Figure 2) were resequenced in 20 HapMap subjects with European ancestry (CEU), as most GWASs for autoimmune diseases have been performed in European cohorts. The number of segregating sites identified in each region is reported in table 2.

**Table 2:** Nucleotide diversity and neutrality tests for the analysed gene regions

Gene	L^a^	P^b^	S^c^	θ_W_^d^		π^e^		Tajima's D			Fu & Li's D*			Fu & Li's F*		
				**value**	**rank^f^**	**value**	**rank^f^**	**value**	**rank^f^**	***p^g^***	**value**	**rank^f^**	***p^g^***	**value**	**rank^f^**	***p^g^***
								
*ZSCAN23*	3.3	CEU	7	4.96	0.44	6.52	0.66	0.87	0.78	0.20	0.51	0.72	0.34	0.73	0.74	0.25
*VARS2*	3.2	CEU	10	7.30	0.69	10.78	0.86	1.43	0.89	0.09	0.24	0.65	0.40	0.73	0.74	0.25
*HLA-DMB*	3.1	CEU	14	10.60	0.91	12.12	0.89	0.45	0.66	0.30	-0.28	0.47	0.60	-0.050	0.54	0.51
*PTPN22*	3.5	CEU	7	4.70	0.44	4.57	0.41	-0.077	0.51	0.49	-0.98	0.23	0.80	-0.81	0.31	0.75
*BAT3*	2.0	CEU	3	3.47	0.21	3.14	0.27	-0.20	0.47	0.57	-0.35	0.46	0.64	-0.36	0.44	0.62
*C6orf47*	2.0	CEU	3	3.59	0.21	5.21	0.48	0.98	0.81	0.18	0.92	0.85	0.19	1.09	0.84	0.13
*IL10*	2.3	CEU	3	2.97	0.15	5.95	0.55	2.12	0.97	0.01	0.90	0.84	0.16	1.47	0.94	0.04
*CDSN/*	4.5	CEU	59	30.80	>0.99	49.42	>0.99	2.17	0.97	0.01	1.40	0.95	0.03	1.98	0.97	0.01
*PSORSC1*																
		YRI	63	32.88	>0.99	46.20	>0.99	1.46	0.99	0.01	1.44	>0.99	<0.01	1.72	>0.99	<0.01
		EAS	64	33.41	>0.99	47.50	>0.99	1.52	0.91	0.08	1.45	0.98	001	1.75	0.98	0.02
*TRIM40/*	9.1	CEU	68	17.51	0.98	26.13	0.99	1.78	0.96	0.03	1.61	0.98	<0.01	1.98	0.97	<0.01
*TRIM10*																
		YRI	64	16.48	0.96	15.74	0.94	-0.16	0.64	0.22	1.07	0.97	<0.01	0.76	0.93	0.02
		EAS	79	20.34	0.99	19.33	0.98	-0.18	0.46	0.31	-0.20	0.46	0.68	-0.23	0.45	0.71
*BTNL2*	3.8	CEU	53	33.04	>0.99	39.75	0.99	-0.33	0.42	0.36	1.32	0.93	0.03	0.88	0.78	0.15
		YRI	63	39.28	>0.99	30.01	>0.99	0.043	0.71	0.18	0.67	0.89	0.06	0.53	0.86	0.07
		EAS	94	58.60	>0.99	47.38	>0.99	-0.70	0.31	0.21	0.95	0.88	0.13	0.43	0.69	0.35
*TAP2*	4.4	CEU	33	17.56	0.98	29.07	0.99	2.27	0.98	0.01	1.59	0.98	0.01	2.16	0.98	<0.01
		YRI	54	28.73	>0.99	34.55	>0.99	0.73	0.94	0.05	1.63	>0.99	<0.01	1.56	0.99	<0.01
		EAS	41	21.81	0.99	32.88	>0.99	1.79	0.97	0.04	1.30	0.96	0.04	1.74	0.98	0.02
TAP2-ctr	2.6	CEU	12	10.71	0.91	12.18	0.89	0.42	0.65	0.32	0.46	0.70	0.29	0.52	0.69	0.18
BTNL2-ctr	2.0	CEU	15	17.47	0.98	16.73	0.96	-0.14	0.50	0.54	1.56	0.97	0.01	1.18	0.90	0.09

^a ^length of analyzed sequenced region (kb);

^b ^sampled population (for each population 40 chromosomes were resequenced);

^c ^number of segregating sites;

^d ^Watterson's theta estimation per site (×10^-4^);

^e ^nucleotide diversity per site (×10^-4^);

^f ^percentile rank relative to a distribution of 238 5kb segments from NIEHS genes;

^g ^*p *value applying demographic coalescent simulations.

Common population genetic tests based on the site frequency spectrum (SFS) include Tajima's D (D_T_) [14] and Fu and Li's D* and F* [15]. D_T _tests the departure from neutrality by comparing two nucleotide diversity indexes: θ_W _[16], an estimate of the expected per site heterozigosity, and π [17], the average number of pairwise sequence nucleotide differences. Positive values of D_T _indicate an excess of intermediate frequency variants and are a signature of balancing selection. Fu and Li's F* and D* are also based on SNP frequency spectra and differ from D_T _in that they also take into account whether mutations occur in external or internal branches of a genealogy [15]. As an empirical comparison, θ_W_, π, as well as D_T_, F* and D* were calculated for 5 kb windows (thereafter referred to as reference windows) deriving from 238 genes resequenced by the NIEHS program in CEU. Additionally, the statistical significance of neutrality tests was evaluated by performing coalescent simulations with a population genetic model that incorporates demographic scenarios [18].

As shown in table 2, no exceptional nucleotide diversity was observed for *ZSCAN23*, *HLA-DMB*, *VARS2*, *PTPN22*, *BAT3*, *C6orf47*, and *IL10*. In line with this observation, summary statistics were consistent with evolutionary neutrality for these gene regions (Table 2). Conversely, the regions we analysed in *CDSN/PSORS1C1*, *TRIM10/TRIM40*, *BTNL2 *and *TAP2 *showed extremely high nucleotide diversity, with both θ_W _and π ranking above the 95^th ^percentile in the distribution of 5kb reference windows.

For *TAP2, CDSN/PSORS1C1*, and *TRIM10/TRIM40 *all tests rejected the null hypothesis of selective neutrality in CEU and ranks of D_T_, F* and D* were higher than the 95th percentile. In the case of *BTNL2*, D*, but not D_T _and F*, was close to statistical significance in the empirical comparison and rejected neutrality when coalescent simulations were performed.

*TAP2 *and *BTNL2 *are located within the classical class II MHC sub-region (Figure 1) and flank a class II HLA gene cluster containing highly polymorphic genes subjected to balancing selection in humans and other primates [19,20]. We therefore wished to verify whether the selection signatures we identified at both genes might be secondary to linkage disequilibrium with HLA class II genes. Thus, intergenic regions flanking the class II HLA gene cluster were also resequenced: as shown in Figure 1, the TAP2-ctr region is telomeric to *TAP2*, while BTNL2-ctr is centromeric to *BTNL2*. These two control regions have very similar divergence to the *TAP2 *and *BTNL2 *regions we analysed (not shown). As reported in table 2, the TAP2-ctr region displayed no exceptional variability and all statistics were consistent with selective neutrality. As for BTNL2-ctr, a high nucleotide diversity was observed but both θ_W _and π were about half the value observed at the *BTNL2 *genic region; only F* displayed a significantly high value. Therefore, high nucleotide diversity at the *TAP2 *locus is not secondary to LD with HLA class II genes. In the case of *BTNL2*, the lower diversity observed at the control compared to the genic region also suggest that the gene is an independent target of balancing selection (see below).

In order to obtain a more comprehensive description of nucleotide diversity, the four regions covering *CDSN/PSORS1C1*, *TRIM10/TRIM40*, *BTNL2*, and *TAP2 *were resequenced in two additional HapMap populations, namely Yoruba (YRI) and East Asians (EAS). For all regions, nucleotide diversity resulted extremely high in YRI and EAS, as well (Table 2). Neutrality tests and empirical comparison with resequenced regions rejected neutrality for these populations at the *CDSN/PSORS1C1 *and *TAP2 *regions. Similar results were obtained for *TRIM10/TRIM40 *in YRI but not in EAS. This latter finding is due to the presence of several singletons that affect SFS-based tests in this population (see below). As for *BTNL2*, the values of SFS-based statistics were not exceptionally high in YRI and EAS.

A hallmark of balancing selection is an excess of polymorphism compared to neutral expectations. Indeed, our data (Table 2) indicate that nucleotide diversity indexes are extremely high for *CDSN/PSORS1C1*, *TRIM10/TRIM40*, *BTNL2 *and *TAP2*. Yet, polymorphism levels also depend on local mutation rates, and under neutral evolution the amount of within- and between-species diversity is expected to be similar at all loci in the genome [21]. The multi-locus HKA test was developed to verify this expectation [22]. We applied a multi-locus MLHKA (maximum-likelihood HKA) test by comparing polymorphisms and divergence levels at the *CDSN/PSORS1C1*, *TRIM10/TRIM40, BTNL2 *and *TAP2 *genomic regions with 16 NIEHS genes resequenced in YRI, CEU and EAS. The results are shown in table 3 and indicate that a significant excess of nucleotide diversity versus divergence is detectable in all populations for all loci. For *TAP2 *and *TRIM10/TRIM40 *the chimpanzee was used for inter-species divergence. Yet, divergence with chimpanzee is unusually low (0.5%) for the *CDSN/PSORS1C1 *region and the reference sequence for orangutan is not available; as for macaque, divergence for *CDSN/PSORS1C1 *(4.8%) is also lower than genome average but not markedly so. Therefore, for *CDSN/PSORS1C1 *the MLHKA test was performed using macaque divergence data. Finally, in the case of *BTNL2*, no reference sequence for chimpanzee is available for the gene region we analysed. Since a partial sequence is available for orangutan, we sequenced the genomic DNA of one *Pongo pygmaeus *(see methods) and used the sequence we obtained for calculation of divergence; therefore the MLHKA test for *BTNL2 *was performed with human/orangutan divergence data.

**Table 3:** MLHKA tests

**Region**	**Pop.^a^**	**MLHKA**	
		
		**k^b^**	***p***
			
*TAP2*	CEU	3.69	1.1 × 10^-3^
	YRI	4.94	6.4 × 10^-5^
	EAS	5.11	3.2 × 10^-5^
			
*CDSN/PSORS1C1*	CEU	4.79	1.6 × 10^-4^
	YRI	3.88	1.6 × 10^-5^
	EAS	5.66	1.6 × 10^-7^
			
*TRIM10/TRIM40*	CEU	3.82	5.5 × 10^-4^
	YRI	2.23	2.1 × 10^-2^
	EAS	4.21	3.9 × 10^-5^
			
*BTNL2*	CEU	5.57	6.4 × 10^-7^
	YRI	5.44	1.7 × 10^-6^
	EAS	10.80	5.6 × 10^-13^

^a ^population

^b^selection parameter (k>1 indicates an excess of polymorphism relative to divergence)

**Figure 3:**
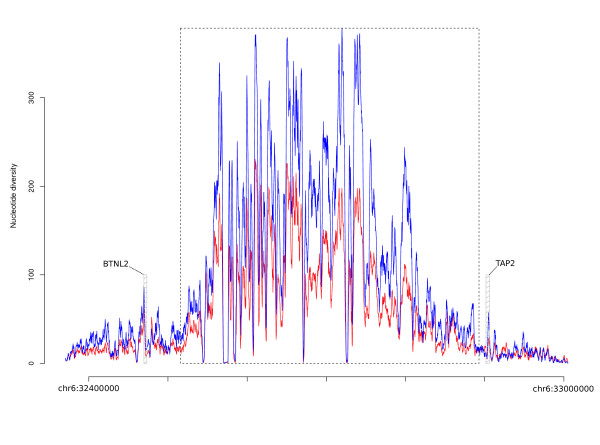
**Sliding window analysis of the genomic region encompassing *BTNL2*, *HLA *class II genes and *TAP2***. Data were obtained from the low-coverage 1000 Genomes Project data and refer to CEU. θ_W _(red) and π (blue) were calculated in 2 kb windows moving with a step of 150 bp. The two region we resequanced in TAP2 and BTNL2 are shown by the shaded boxes. The hatched region indicates the genomic portion occupied by HLA class II genes.

We next took advantage of the availability of data from the 1000 Genomes Pilot project [23] to validate our results in a larger poulation sample. The low-coverage 1000 Genomes approach, which generated whole genome sequencing data of 179 individuals with different ancestry (YRI, CEU and EAS), is estimated to have relatively low power to detect singleton SNPs or rare variants [23]. Thus, an empirical comaprison is needed to evaluate whether selected gene regions display diversity indexes or SFS-based staistics that are exceptionally high (i.e. that reject neutral expectations). To this aim we randomly selected 2,000 human genes and, from each of them, one 5 kb window was extracted. We next calculated diversity indexes and SFS-based statistics, as in table 2. All results were confirmed using this approach (Additional file 2). Finally, we used the 1000 Genomes data to perform a sliding-window analysis of an extended genomic regions covering *BTNL2*, the MHC class II gene cluster, and *TAP2 *plus flanking regions. As shown in Figure 3, two peaks of diversity were observed at the *BTNL2 *and *TAP2 *regions we resequenced. These are separated from the MHC class II region (showing extreme diversity, as expected) by segments showing lower values of both θ_W _and π, suggesting that they represent independent selection targets.

### Haplotype analysis and TMRCA estimates

**Figure 4:**
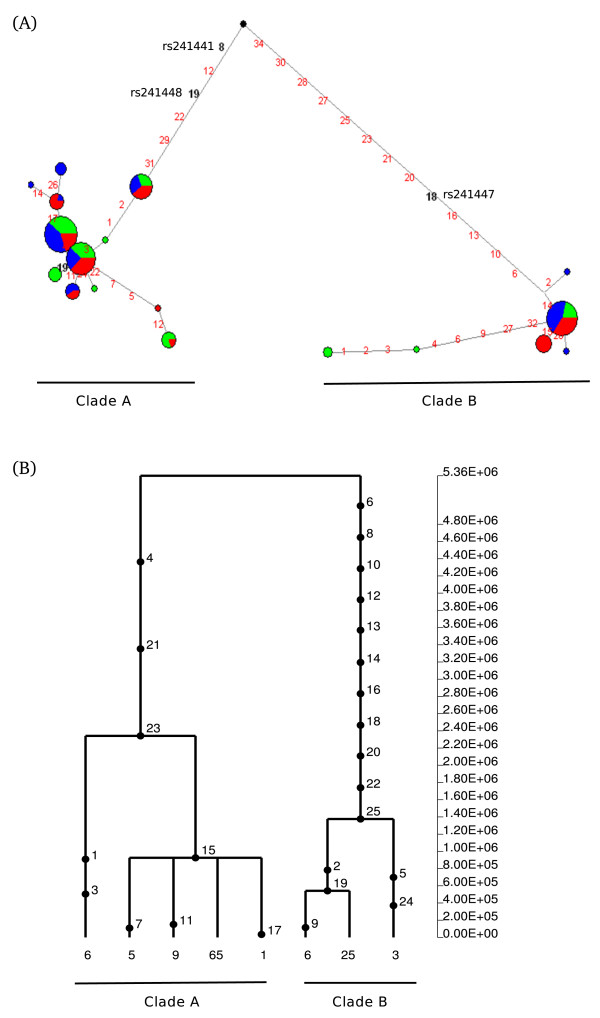
**Haplotype genealogy of the analysed *TAP2 *region**. (A) Network analysis. Each node represents a different haplotype, with the size of the circle proportional to frequency. Nucleotide differences between haplotypes are indicated on the branches of the network. Circles are colour-coded according to population (green: YRI, blue: CEU, red: EAS). The most recent common ancestor is also shown (black circle). The relative position of mutations along a branch is arbitrary. (B) GENETREE analysis. Mutations are represented as black dots and named for their physical position along the regions. The absolute frequency of each haplotype is also reported. Note that mutation numbering does not correspond to that reported in (A).

Further insight into the evolutionary history of a gene region can be gained by inferring haplotype genealogies. This has both a descriptive purpose (i.e. showing the relationship among alleles and their distribution in human populations) and can be used to test for selection. In particular, balancing selection is expected to result in two or more major haplotype clades with a deep coalescence time (TMRCA, time to the most common recent ancestor). In fact, while neutrally evolving autosomal loci have TMRCAs ranging from 0.8 to 1.5 million years (MY) [24], gene regions under balancing selection may show coalescence times dating back more than 4 MY [25-27]. Here we constructed haplotype genealogies using two approaches: a neighbour-joining network and a maximum-likelihood coalescent method implemented in GENETREE. This latter assumes an infinite-site model without recombination, requiring the removal of variants and haplotypes that violate these assumptions. In order to obtain more reliable trees, we selected sub-regions based on LD for those genomic regions showing high recombination rates. Thus, for *TAP2 *we used data from a 1.9 kb region with relatively high LD (Additional files 3 and 4). This region does not encompass the opposite risk SNP but includes a set of markers (rs241448, rs241447 and 241441, variants 19, 18 and 8, respectively in the network, Figure 4A) previously known to identify the two haplotypes that generate alternatively spliced isoforms [11]. As it is evident from both the network and GENETREE analysis (Figure 4), the *TAP2 *genealogy is split into two major haplotype clades with an estimated time to the most recent common ancestor (TMRCA) of ~5.36 MY. The two clades differ at several variants including those affecting *TAP2 *splicing.

**Figure 5:**
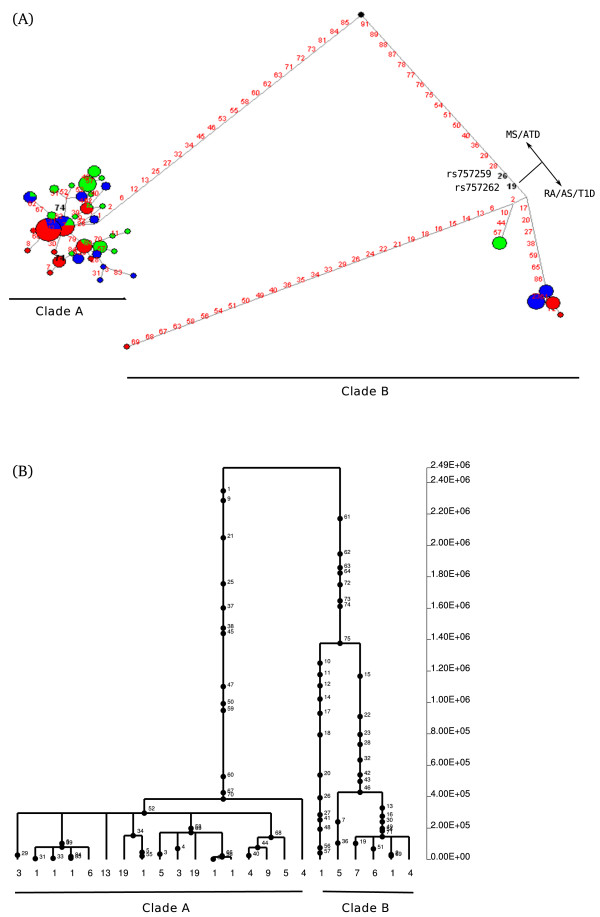
**Haplotype genealogy of the analysed *TRIM10/TRIM40 *region**. Legend is as for figure 3. In addition an indication is given as to which haplotypes predispose to RA/AS/T1D or MS/ATD.

Similarly, due to extensive recombination, a sub-region with stronger LD (Additional files 3 and 4) was analysed in the case of *TRIM10/TRIM40*. The rs2517646 variant (table 1) lies outside this region, whereas the second variant with an opposite risk profile (rs757262) is located on the major branch leading to clade B (Figure 5A). As evident in both the network and GENETREE analyses a single, highly divergent haplotype is observed in EAS (Figure 5); although several positions along the branch are recurrent and possibly originate from recombination between the two major clades or gene conversion, 13 SNPs are specific to this haplotype and represent singletons in EAS, therefore affecting SFS-based statistics (Table 2).

**Figure 6:**
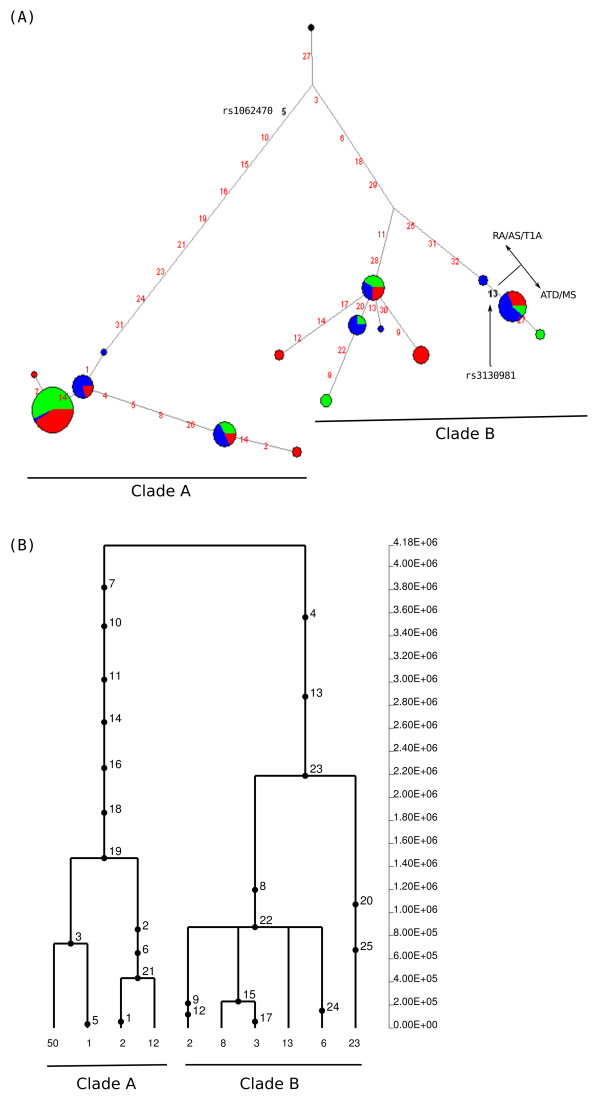
**Haplotype genealogy of the analysed *CDSN/PSORS1C1 *region**. Legend is as for figure 4.

In the case of *CDSN/PSORS1C1*, again we selected a 2 kb region of relative LD (Additional files 3 and 4) which includes one of the two variants with opposite risk-profile (variants 13 in the network, Figure 6A). The two major clades of the genealogy have a TMRCA of 4.18 MY and clade B is split into two main haplogroups that coalesce at 2.2 MY (Figure 6B), possibly suggesting a multiallelic balancing selection regime. It is worth noting that, for the same reasons reported above, the coalescence time was calibrated on the basis of a mutation rate calculated from human/macaque divergence. The opposite-risk SNP defines a subset of haplotypes in clade B (Figure 6A).

**Figure 7:**
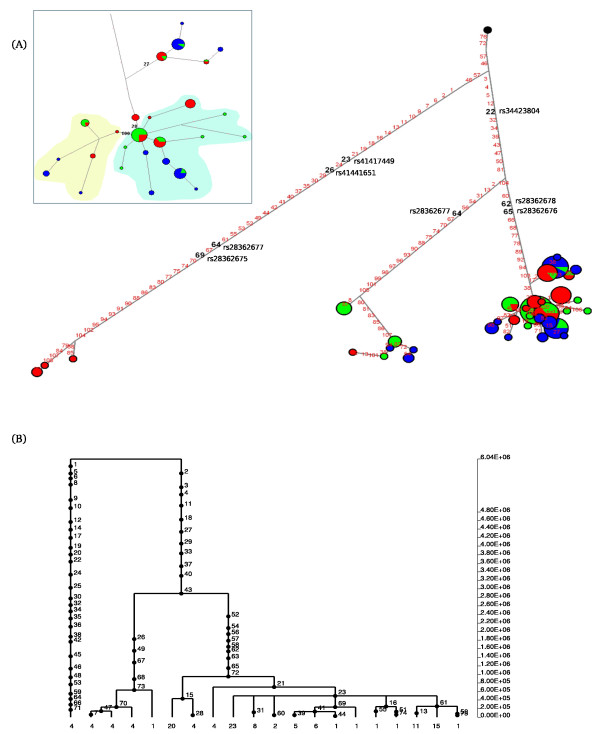
**Haplotype genealogy of the analysed *BTNL2 *region**. Legend is as for figure 3. In (A) positions in bold refer to the following SNPs: 22, rs34423804 (Val189Asp); 23, rs41417449 (Met201Val); 26, rs41441651 (Asp242Asn); 62, rs28362678 (Pro285Leu); 64, rs28362677 (Met286Ile); 65, rs28362676 (Pro299Gln); 69, rs28362675 (Gly360Cys). An enlargement of the major haplotype cluster is also shown (left-upper panel): susceptibility haplotypes for ATD and MS are circled in yellow and cyan, respectively. SNPs numbering is as follows: 27, rs9268480; 28, rs2076530; 100, 3129953.

Finally, in the case of *BTNL2*, the gene portion we analysed is in relative tight LD and the network and GENETREE analyses were performed over the entire region. Three major haplotype clades are evident with the most distantly related haplotype cluster being present in EAS only (Figure 7).

Conversely, chromosomes from all populations contribute to the two remaining clades, although with extremely different frequency. The TMRCA for the whole genealogy amounts to ~ 6 MY, while the two more closely related clades coalesce at ~ 2.8 MY (Figure 7B). The branches leading to the two lower-frequency clades share some variants and their relatively close physical proximity suggests that these apparent homoplasies are due to a recombination or gene conversion event. The two variants with opposite effect are not located on the major branches of the genealogy but define haplotye subsets within the major clade (Figure 7A).

This haplotype genealogy explains the results we obtained for SFS-based statistics in this region; the presence of two (CEU and YRI) or three (EAS) deeply separated haplotype clades introduces a high number of polymorphic sites (resulting in high nucleotide diversity indexes). Yet, clade 2 haplotypes are present at low frequency in all populations and the same applies to clade 1 in EAS. Therefore, the frequency spectrum is not markedly skewed towards intermediate frequency alleles and, consequently, most SFS-based statistics fail to reject the null hypothesis of neutrality.

Nonetheless, the haplotype genealogy and TMRCAs we obtained are not consistent with neutral evolution in an unstructured population but rather suggest the action of balancing selection and/or a possible contribution of archaic population structure in Asia. An alternative possibility is that, as discussed below, this pattern is due to LD with HLA class II genes.

## Discussion

Autoimmune diseases are common in industrialized societies, collectively reaching a prevalence of 5% in populations with European ancestry [28]. Epidemiological studies have indicated that the incidence of these disorders has been steadily increasing during the last decades in the industrialized world. Therefore, much scientific debate has addressed the role of human evolutionary history and adaptation in shaping the genetic predisposition to the development of autoimmunity [29]. For any single autoimmune condition, more than 50% of the disease risk is heritable [30] and GWASs have unveiled the role of several common risk variants, possibly reflecting an allelic architecture for autoimmune disease that matches a "common variant/common disease" model more closely than observed for other traits [30]. From the evolutionary perspective, this raises interesting questions on the forces responsible for the maintenance of disease alleles in populations (reviewed in [29]). Since the evolutionary history of autoimmune-related alleles is only beginning to be investigated, our knowledge is still relatively limited in this field. Specifically, a subset of risk alleles for CD and UC has previously been shown to have evolved in response to pathogen-driven selective pressures, the underlying scenario for some of them being balancing selection [12]. More recently, several disease alleles for autoimmune conditions were reported to display signatures of directional selection in favour of the risk alleles [31]. Finally, two common variants for T1D, an early-onset, potentially lethal disease, have been described as neutrally evolving [32]. Albeit limited to a small number of variants, these data do not support the notion whereby autoimmune phenomena have acted as a selective pressure strong enough to affect the frequency distribution of risk alleles, although some authors have speculated that balancing selection at innate immunity genes might stem from a tuning of response to pathogens and to self [33]. The identification of a set of variants with an opposite risk profile for different autoimmune conditions prompted the speculation that these variants might be targets of balancing selection possibly deriving from antagonistic pleiotropy. Therefore the question is: if neither allele can be considered medically favourable, are they both evolutionary beneficial under specific circumstances? Our data indicate that 4 out of ten gene regions we analysed have been subjected to balancing selection. Additionally, *IL18RAP *has previously been indicated as a possible selection target [12]. Similarly, a signature of balancing selection has previously been described at the promoter region of *IL10 *[13], suggesting that the selected variant might be different from the opposite risk allele which is located downstream the transcription end site. The evidences we report herein for the *TAP2*, *TRIM10/TRIM40*, and *CDSN/PSORS1C1 *gene regions are all consistent with the hypothesis whereby genetic variability is maintained at these loci by long-standing balancing selection. In the case of *BTNL2 *and *TAP2*, we addressed the possibility that LD with HLA class II genes influences the results we obtained, as genetic hitch-hiking can potentially affect neutral diversity over long genomic regions. Yet, the sliding window analysis of nucleotide diversity across the region encompassing these two genes and HLA class II loci indicated that peaks at *BTNL2 *and *TAP2 *are flanked by regions with lower diversity, suggesting that these two genes represent independent (from class II loci) selection targets.

Conversely, we identified no selection signature for the remaining 6 genes, namely *ZSCAN23*, *PTPN22*, *HLA-DMB*, *VARS2*, *C6orf47*, and *BAT3*. In all these cases nucleotide diversity was within average values and no test significantly deviated from the null hypothesis of neutral evolution. One possibility is that opposite risk SNPs in these regions do not represent causal variants but genetic markers and, therefore, that natural selection might be acting in a region different from the one we resequenced. Yet, this is unlikely to be the case for *PTPN22*, as the opposite risk variant has been shown to be functional. The derived 602W allele, which segregates at low frequency in Caucasian populations and is extremely rare outside Europe, confers to the phosphatase a stronger ability to inhibit the T cell receptor signalling pathway [34]; this allele has been associated with T1D, RA and other autoimmune manifestations [35]. Conversely, the ancestral allele has been associated with a higher risk of developing CD. Our analysis of the region carrying the R602W variant did not unveil any molecular signature of natural selection, as all tests were consistent with neutrality. Nonetheless, the power of most tests is strongly influenced by the timing and strength of the selective pressure; it has been suggested that the 602W allele has risen in frequency in some European populations as a result of natural selection, as its frequency seems to increase with latitude [35]. An extremely recent selective event in these populations would not be detected using our approach.

The four targets of balancing selection we identified in this study are all located within the xMHC and have different molecular functions. *TAP2 *is a central component of the antigen processing pathway; the protein products of *TAP1 *and *TAP2 *interact to form a transporter complex (TAP) that translocates antigenic peptides to the endoplasmic reticulum (ER) where loading onto MHC class I molecules occurs. Therefore, inhibition of TAP has been exploited by different viruses as an immune evasion strategy (reviewed in [36]). Specifically, proteins encoded by herpesviruses (HSV), human cytomegalovirus (HCMV) and Epstein-Barr virus can block TAP function, limit the supply of peptides to the endoplasmic reticulum and therefore inhibit MHC class I maturation. Interestingly, the ability of HSV- and HCMV-encoded proteins to block TAP function is species-specific, suggesting that viral products have co-evolved with the TAP molecules of their host species. This observation also suggests that *TAP1 *and *TAP2 *may be subjected to a selective pressure exerted by viruses to avoid binding of inhibitory proteins. The *TAP2 *gene portion we analysed herein is subjected to a haplotype-specific alternative splicing event that results in two molecular forms differing at the C-terminus; our data show that the two haplotypes associated with alternative splicing are maintained by balancing selection. Whether the two protein products are differentially sensitive to viral inhibitors is presently unknown, but a previous report has indicated that they display marked differences in the translocation efficiency of specific peptides. This difference may have an effect on the susceptibility to specific viral infections and the Ala665Thr variant (rs241447), which is associated with alternative splicing, and located on the major branch leading to clade b (Figure 4A) has been associated to altered susceptibility to HIV-1 infection [37].

Two genes encoding ER aminopeptidases that trim peptides translocated by TAP have recently been shown to be subjected to long-standing balancing selection with polymorphic variants conferring resistance against HIV-1 [26]. It is therefore tempting to speculate that maintenance of genetic diversity at genes in the antigen processing pathway derives from the differential activity of diverse alleles for specific peptides, eventually leading to distinctive repertoires of antigens presented to CD8^+ ^T cells and, possibly, altered susceptibility to specific infections. Nonetheless, it is worth noting that the allele with opposite risk profile (rs10484565) has a low MAF (frequency of minor allele < 0.10) in all populations and our data suggest that it is not (and is not in linkage with) the balancing selection target.

*TRIM10 *and *TRIM40 *code for two members of a large family of tripartite motif-containing (TRIM) proteins. While several TRIM proteins have been shown to act as antiviral factors, the role of TRIM10 and TRIM40 is virtually unknown, although TRIM10 may have a role in erythropoiesis [38]. A recent GWAS for host genetic factors involved in HIV control identified a SNP (rs9468692) located in 3'UTR of *TRIM10*, within the region we analysed. Our resequencing data indicate that this SNP is triallelic with T/G alleles segregating in CEU and YRI, and A/G in EAS. This is clearly shown in the network analysis: variant 74 defines two different haplotype clusters one containing African and European chromosomes and the other Asian haplotypes only. The variant involves a CpG dinucleotide suggesting that the triallelic status is determined by a two-hit mutation process on different haplotypic backgrounds and involving deamination of 5-methyl cytosine in the case of the A/G alleles. While the description of a triallelic variant might have a relevance for future association studies in populations with different ancestry, its location in the network suggests that the site is neutrally evolving. Conversely, the Thr183Met variant (rs757262, variant 19 in Figure 5A) with opposite risk profile (Table 1) is in strong LD with another nonsynonymous variant (Glu215Lys, rs757259, variant 26 in Figure 5A) and both SNPs separate the two major haplotype clades, suggesting that they may represent the selection target(s). The limited knowledge on the biological function of *TRIM10 *and *TRIM40 *does not allow extensive speculation on the selective pressure responsible for maintaining nucleotide diversity at these genes and further functional studies will be required to determine whether it is virus-driven or not.

The third gene region which we found to be subjected to balancing selection covers part of *CDSN *and *PSORS1C1*. A previous work using a different dataset has also suggested that CDSN might be a balancing selection target [39]. *CDSN *and *PSORS1C1 *the two genes are transcribed in the opposite direction and the whole coding region of corneodesmosin (*CDSN*) is comprised within the first intron of *PSORS1C1 *(also known as *SEEK1*). Both transcripts are widely expressed http://biogps.gnf.org and have been associated with psoriasis in several studies. Specifically, *CDSN *is up-regulated in psoriatic lesisons [40] and one psoriasis-associated allele (rs1062470, variant 5 in Figure 6A) affects the binding of an unknown cellular factor, resulting in increased transcript stability [40]. This SNP is located on the network branch separating the two major haplotype clades, suggesting that it may represent the selected variant. Similarly, one of the two SNPs (rs3130981, Asp527Asn) with an opposite effect on autoimmune diseases defines a major haplotype group within clade b, indicating that it may represent (or be in linkage with) a selection target (in a multiallelic selection regime).

The role of the protein product of *PSORS1C1 *is presently unknown, although one SNP in *PSORS1C1 *immediately downstream the region we analysed has been associated through GWAS with white blood cell counts [41] and a second downstream variant with delayed AIDS progression [42]. Conversely the role of *CDSN *is well studied as the gene encodes corneodesmosin, an adhesive protein which participates in the stabilization of corneodesmosomes in the skin and other cornified squamous epithelia. Loss of coreneodesmosin in humans results in generalised peeling skin disease, a condition characterized by skin barrier defects, pruritus and atopy, with patients also showing *Staphylococcus aureus *superinfections [43]. Mice lacking *CDSN *display a severe phenotype and the skin barrier defect is accompanied by a 10-fold increase in transepithelial water loss. These observations highlight the central role played by the epidermal barrier in protection from infections and in water homeostasis, two processes that are though to have been targeted by natural selection during human evolutionary history. Therefore, SNPs in *CDSN *may have been maintained by balancing selection due to their modulation of the skin barrier properties. Still, the observation that humans with genetic defects in *CDSN *also display food allergies [43] suggests that the protein may play a role in processes unrelated to skin function.

Finally, *BTNL2 *encodes a butrophilin-like, widely expressed protein with still poorly understood function. Experiments in mice have suggested that *BTNL2 *might function as a negative co-stimulatory molecule with inhibitory activity on T cell activation [44,45]. In particular, a possible modulatory role for the protein in intestine inflammation has been proposed in this animal model, in line with the observation that a SNP (rs9268480) in the region we analysed has also been associated with UC [46].

The haplotype genealogy of the *BTNL2 *region we resequenced is peculiar with one deeply separated clade limited to EAS subjects. Previous studies have suggested that such tree topologies might originate from admixture of anatomically modern humans with archaic hominin populations (reviewed in [47]) rather than by a selective force; yet, a TMRCA > 5 MY may be unlikely even under ancient admixture scenarios [47]. Therefore, we suggest that the distantly related clade is maintained by a selective process acting on *BTNL2 *or on the nearby class II MHC loci. As reported above, the coalescence time of the two more closely related clades is also deep and several nonsynonymous variants are located on the major branches (Figure 7A). Specifically, 3D modelling of BTNL2 [48] indicated that the Pro285Leu, Met286Ile and Pro299Gln variants are all located in close physical proximity within the IgC domain and adjacent to cystein residues involved in disulfide bonding. These variants are postulated to be functional [48] and can be regarded as potential selection targets. Conversely both the opposite risk alleles and the UC susceptibility variant identify haplotype subsets within the major clade (Figure 7A).

Therefore, even in those regions where we did identify balancing selection signatures, the opposite risk alleles do not always represent (or are in tight linkage with) the selected variants. Rather, our data suggest that, with the exclusion of rs757262 and rs3130981 in *TRIM40 *and *CDSN*, opposite risk SNPs are likely to have accumulated as neutral variants, possibly maintained through hitch-hiking with the balanced polymorphisms. An alternative possibility is that, as in the case of the gene regions that we described as neutrally evolving, weaker and more recent selective events have been maintaining the opposite risk alleles. In this respect it is worth noting that the haplotype structure of the gene regions we analysed is often complex, raising the possibility that the opposite risk profile identified for some of these SNPs is secondary to association with another causal variant which is not analysed in the association study. In the case of *BTNL2*, for example, we were able to include two opposite risk alleles in the network analysis; based on the risk profiles of variants 28 and 100 (rs2076530 and rs3129953, respectively, Table 1) we were able to identify a set of haplotypes that should confer increased risk for ATD (they carry 2 predisposing alleles) and a group of haplotypes that predispose to MS (again with two risk alleles) (Figure 7A). This means that rs2076530 and rs3129953 may simply define haplotype subsets that carry causal variants for MS and ATD but when these variants are not typed in the association study, the haplotype structure is such that the derived allele of at position 100 will be under-represented among MS patients for example, resulting in an apparent protective effect. A similar situation might apply to *CDSN*, as well (Figure 6A).

## Conclusion

Data herein indicate that balancing selection is common within the xMHC region and involves several non-HLA loci. Yet, the evolutionary history of most SNPs with an opposite effect for autoimmune diseases is consistent with evolutionary neutrality. We suggest that variants with an opposite effect on autoimmune diseases should not be considered a distinct class of disease alleles from the evolutionary perspective. Moreover, in a few cases, the opposite effect on distinct diseases may derive from complex haplotype structures in regions where genetic diversity is high and typing in association studies only captures limited information.

## Methods

### HapMap samples and sequencing

Human genomic DNA from HapMap subjects was obtained from the Coriell Institute for Medical Research. All analysed regions were PCR amplified and directly sequenced; primer sequences are available upon request. PCR products were treated with ExoSAP-IT (USB Corporation Cleveland Ohio, USA), directly sequenced on both strands with a Big Dye Terminator sequencing Kit (v3.1 Applied Biosystems) and run on an Applied Biosystems ABI 3130 XL Genetic Analyzer (Applied Biosystems). Sequences were assembled using AutoAssembler version 1.4.0 (Applied Biosystems), and inspected manually by two distinct operators. Orangutan (*Pongo Pygmaeus*) genomic DNA (Cell line name: EB185_JC) was obtained from European Collection of Cell Cultures. In order to fill gaps in the reference sequence, we PCR-amplified two genomic region using the following primer sets: F1 (5'-TGGAGTGCAGTGGCATGATC-3')/R1 (5'-TCAGTCTGCCCTCGTCAATG-3') and F2 (5'-CTTGTCAGAGTGGGAGAAGAT-3')/R2 (5'-CTCAGAGGAGTAGAATCCCTG-3'). The PCR products F1/R1 and F2/R2 were sequenced with seqF1 (5'-CTCGTCAGAGTGGGAGAAGAT-3') and R2, respectively.

### Data retrieval and haplotype construction

Information on SNPs identified in GWAS were retrieved from the National Human Genome Institute website (A Catalog of Published Genome-Wide Association Studies; http://www.genome.gov/26525384) updated to September the 1st, 2010. Data on gene expression in different human tissues were derived from the BioGPS website http://biogps.gnf.org. Genotype data for 5 kb regions from 238 resequenced human genes were derived from the NIEHS SNPs Program web site http://egp.gs.washington.edu. In particular we selected genes that had been resequenced in populations of defined ethnicity including Europeans (CEU), Yoruba (YRI) and East Asians (EAS) (NIEHS panel 2).

Haplotypes were inferred using PHASE version 2.1 [49,50]. Linkage disequilibrium analyses were performed using the Haploview (v. 4.1) [51] and blocks were identified through an algorithm implemented in the software.

Data from the Pilot 1 phase of the 1000 Genomes Project were retrieved from the dedicated website http://www.1000genomes.org/. Low coverage SNP genotypes were organized in a MySQL database. A set of programs was developed to retrieve genotypes from the database and to analyse them according to selected regions/populations. These programs were developed in C++ using the GeCo++ [52] and the LibSequence [53] libraries. Genotype information was obtained for all analysed region and for 2,000 regions (5kb in size) randomly derived from an equal number of RefSeq genes.

### Statistical analysis

Tajima's D [14], Fu and Li's D* and F* [15] statistics, as well as diversity parameters θ_W _[16] and π [17] were calculated using *libsequence *[53]. Calibrated coalescent simulations were performed using the *cosi *package [18] and its best-fit parameters for YRI, EU and EAS populations with 10,000 iterations. Briefly, this model supposes a series of population size changes occurring at each population, along with migratory events, with intensity and duration estimated by fitting demographic parameters with genome-wide real data (see[18] for further details including parameters of the best-fitting model). A major advantage of this model is that it can generates simulated data closely resembling empirical data, rather than accurately infer historical scenarios for human populations. Coalescent simulations were performed conditioned on the local mutation rate, estimated from the number of fixed differences with an out-group species, and on the local recombination rate, retrieved by UCSC Genome Browser tables (ucsc.genome.gov). The maximum-likelihood-ratio HKA test was performed using the MLHKA software [22], as previously proposed [54]. Briefly, 16 reference loci were randomly selected among NIEHS loci shorter than 20 kb that have been resequenced in the 3 populations; the only criterion was that Tajima's D did not suggest the action of natural selection (i.e. Tajima's D is higher than the 5^th ^and lower than the 95^th ^percentiles in the distribution of NIEHS genes). The reference set was accounted for by the following genes: *VNN3, PLA2G2D, MB, MAD2L2, HRAS, CYP17A1, ATOX1, BNIP3, CDC20*, *NGB, TUBA1, MT3, NUDT1, PRDX5, RETN *and *JUND*. The chimpanzee, orangutan or macaque sequence was used as the out-group as detailed in the text.

### Haplotype analysis and TMRCA calculation

Median-joining networks to infer haplotype genealogy was constructed using NETWORK 4.5 [55]. Estimate of the time to the most common ancestor (TMRCA) derived from application of a maximum-likelihood coalescent method implemented in GENETREE [56,57]. Again, the mutation rate μ was obtained on the basis of the divergence between human and chimpanzee or orangutan or macaque and under the assumption both that the species separation occurred 6, 13 and 23 MY ago, respectively [58] and of a generation time of 25 years. The migration matrix was derived from previous estimated migration rates [18]. Using this μ and θ maximum likelihood (θ_ML_), we estimated the effective population size parameter (N_e_). With these assumptions, the coalescence time, scaled in 2N_e _units, was converted into years. For the coalescence process, 10^6 ^simulations were performed. Details on the GENETREE analyses are available in Additional file 5.

All calculations were carried out in the R environment [59].

## Abbreviations

SNP: Single Nucleotide Polymorphism; MY: million years; YRI: Yorubans; CEU: Europeans; EAS: East Asians; TMRCA: Time to the Most Recent Common Ancestor; D_T_, Tajima's D; GWAS: genome-wide association study; CD: Crohn's disease; UC: ulcerative colitis; MS: multiple sclerosis; T1D: type 1 diabetes; RA: rheumatoid arthritis; ATD: autoimmune thyroid disease; AS: and ankylosing spondylitis; SLE: systemic lupus erythematosus.

## Authors' contributions

RC and SR performed all resequencing experiments and analysed the data; MF and UP performed most population genetics analyses; MS, MF, RC, GPC and UP analysed and interpreted the data; NB participated in the study coordination. MS and MC wrote the paper. MS and MC conceived and coordinated the study. All authors read and approved the final manuscript.

## Supplementary Material

Additional file 1 **Schematic representation of the gene regions we resequenced in *ZSCAN23*, *HLA-DMB*, *VARS2*, *C6orf47, BAT3, PTPN22 *and *IL10***. Click here for file

Additional file 2 **Nucleotide diversity indexes and summary statistics calculated from the low-coverage 1000 Genomes data**. Click here for file

Additional file 3 **LD map of the genomic regions we resequenced in *TAP2 *region (chr6:32901567-32905985), *TRIM40/TRIM10 *region (chr6:30221583-30230714), *CDSN/PSORS1C1 *region (chr6:31188857-31193360)**. Click here for file

Additional File 4 **Nucleotide diversity estimates and summary statistics for the sub-regions included and excluded from GENETREE analysis**. Click here for file

Additional file 5 **Table with details on GENETREE analyses**. Click here for file
