# Supplementary material for: Balancing selection is common in the extended MHC region but most alleles with opposite risk profile for autoimmune diseases are neutrally evolving
Source: BMC Evol Biol. 2011 Jun 17;11:171. doi: 10.1186/1471-2148-11-171 (PMC3141431; doi:10.1186/1471-2148-11-171)

Additional file 1. Schematic representation of the gene regions we resequenced in *ZSCAN23, HLA-DMB, VARS2, C6orf47, BAT3, PTPN22* and *IL10*.

Transcribed regions are shown in grey; different transcripts either from the same or from different genes are shown separately. The direction of transcription is indicated by the arrows. The location of SNPs with opposite risk profile is reported.

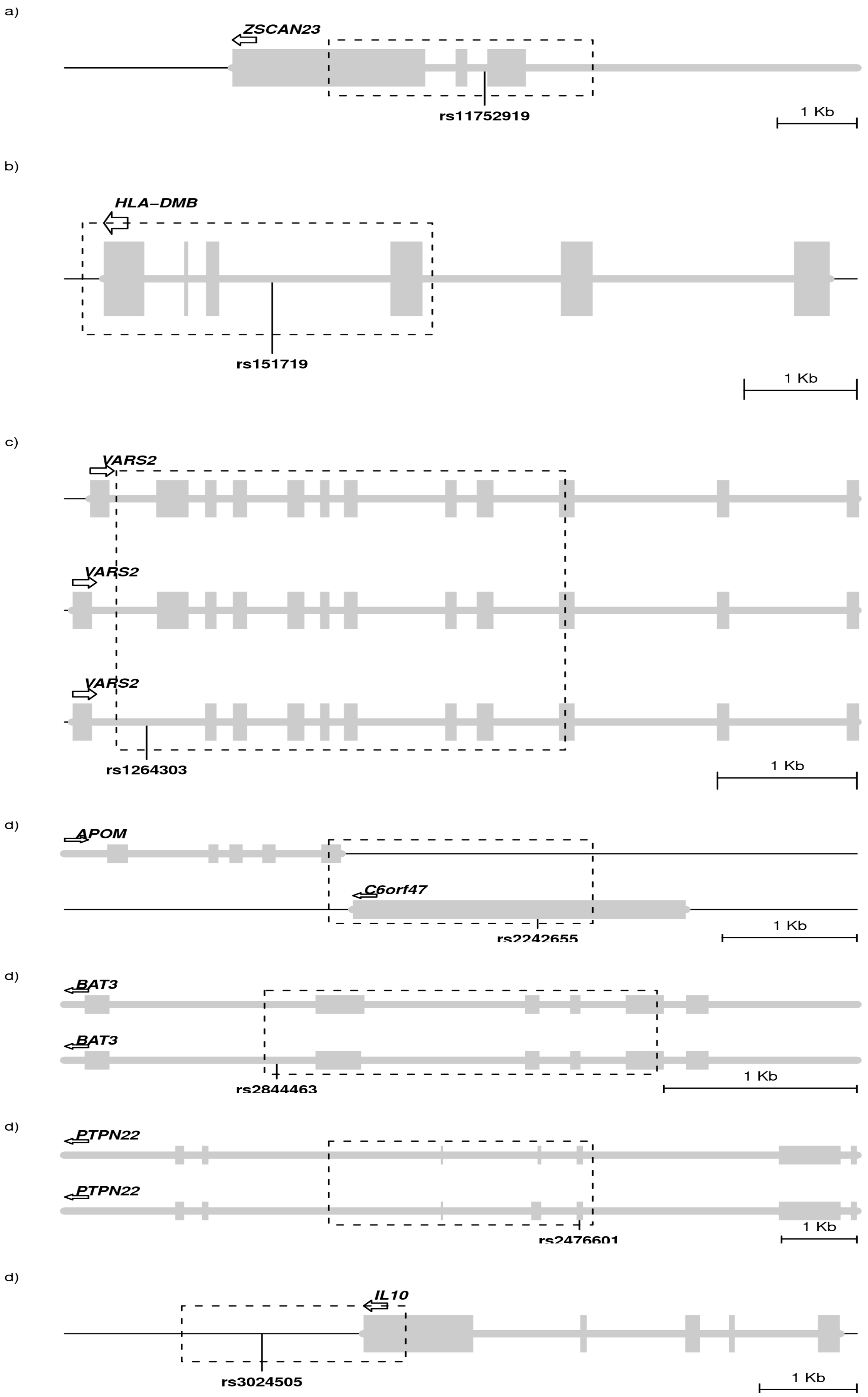

Supplement: Additional file 1 — Schematic representation of the gene regions we resequenced in ZSCAN23, HLA-DMB, VARS2, C6orf47, BAT3, PTPN22 and IL10. [file 1471-2148-11-171-S1.PDF]
