# Supplementary material for: Balancing selection is common in the extended MHC region but most alleles with opposite risk profile for autoimmune diseases are neutrally evolving
Source: BMC Evol Biol. 2011 Jun 17;11:171. doi: 10.1186/1471-2148-11-171 (PMC3141431; doi:10.1186/1471-2148-11-171)
Supplement: Additional file 2 — Nucleotide diversity indexes and summary statistics calculated from the low-coverage 1000 Genomes data. [file 1471-2148-11-171-S2.PDF]

Additional File 2. Nucleotide diversity and neutrality tests using the low-covearge 1000 Genomes Project data.

Gene	L[a]	P[b]	S[c]	θ_W[d]		π[e]		Tajima's D		Fu & Li's D*		Fu & Li's F*	
				value	rank[f]	value	rank[f]	value	rank[f]	value	rank[f]	value	rank[f]
ZSCAN23	3.3	CEU	10	5.65	0.78	5.18	0.61	-0.21	0.27	1.36	0.85	0.96	0.62
VARS2	3.2	CEU	13	7.58	0.91	14	0.96	2.22	0.94	1.50	0.92	2.10	0.96
HLA-DMB	3.1	CEU	14	8.42	0.93	11.5	0.92	0.98	0.67	-0.22	0.20	0.27	0.32
PTPN22	3.5	CEU	10	5.33	0.75	5.55	0.65	0.11	0.36	0.64	0.48	0.54	0.43
BAT3	2	CEU	3	2.80	0.34	2.93	0.35	0.083	0.36	-0.63	0.11	-0.47	0.14
C6orf47	2	CEU	4	3.73	0.48	4.73	0.56	0.51	0.50	-0.30	0.18	-0.045	0.23
IL10	2.3	CEU	4	3.24	0.41	5.87	0.68	1.56	0.82	0.93	0.65	1.33	0.77
CDSN / PSORSC1	4.5	CEU	70	29	>0.99	56	>0.99	2.96	0.99	1.65	0.97	2.65	>0.99
CDSN / PSORSC1	4.5	YRI	75	31.2	>0.99	50.8	>0.99	2.01	0.98	2.22	>0.99	2.56	>0.99
CDSN / PSORSC1	4.5	EAS	67	27.8	>0.99	47.5	>0.99	2.25	0.87	2.35	>0.99	2.77	0.99
TRIM40 / TRIM10	9.1	CEU	70	14.3	0.99	23.6	0.99	2.06	0.91	0.93	0.65	1.69	0.89
TRIM40 / TRIM10	9.1	YRI	80	16.5	0.99	16.7	0.96	0.054	0.46	1.93	0.98	1.35	0.81
TRIM40 / TRIM10	9.1	EAS	68	13.9	>0.99	21.9	0.99	1.82	0.77	2.35	>0.99	2.55	0.98
BTNL2	3.8	CEU	53	26	>0.99	37.1	>0.99	1.33	0.77	1.80	0.99	1.92	0.94
BTNL2	3.8	YRI	62	30.5	>0.99	36.1	>0.99	0.58	0.70	1.32	0.69	1.21	0.74
BTNL2	3.8	EAS	115	56.5	>0.99	74.3	>0.99	1.03	0.52	2.42	>0.99	2.17	0.93
TAP2	4.4	CEU	44	18.7	>0.99	30.7	>0.99	1.99	0.90	1.90	>0.99	2.32	0.99
TAP2	4.4	YRI	57	24.2	>0.99	28.9	>0.99	0.61	0.72	2.27	>0.99	1.89	0.96
TAP2	4.4	EAS	37	15.7	>0.99	30.5	>0.99	2.87	0.96	2.07	>0.99	2.88	>0.99

[a] length of analyzed sequenced region (kb);
[b] sampled population (number of chromosomes is 120 for CEU and EAS, 118 for YRI);
[c] number of segregating sites;
[d] Watterson's θ estimation per site (x 10^{-4});
[e] nucleotide diversity per site (x 10^{-4});
[f] percentile rank relative to a distribution of 5 kb regions deriving from 2,000 randomly selected RefSeq genes.
