# Supplementary material for: Balancing selection is common in the extended MHC region but most alleles with opposite risk profile for autoimmune diseases are neutrally evolving
Source: BMC Evol Biol. 2011 Jun 17;11:171. doi: 10.1186/1471-2148-11-171 (PMC3141431; doi:10.1186/1471-2148-11-171)

Additional File 3. LD map of the genomic regions we resequenced.
The regions we used for the network and GENETREE analyses are indicated by the arrows. Gene regions are as follows: (A) *TAP2* region (chr6:32901567-32905985); (B) *TRIM40/TRIM10* region (chr6:30221583-30230714); (C) *CDSN/PSORS1C1* region (chr6:31188857-31193360). Coordinates refer to the NCBI Build 36.1.

(A)

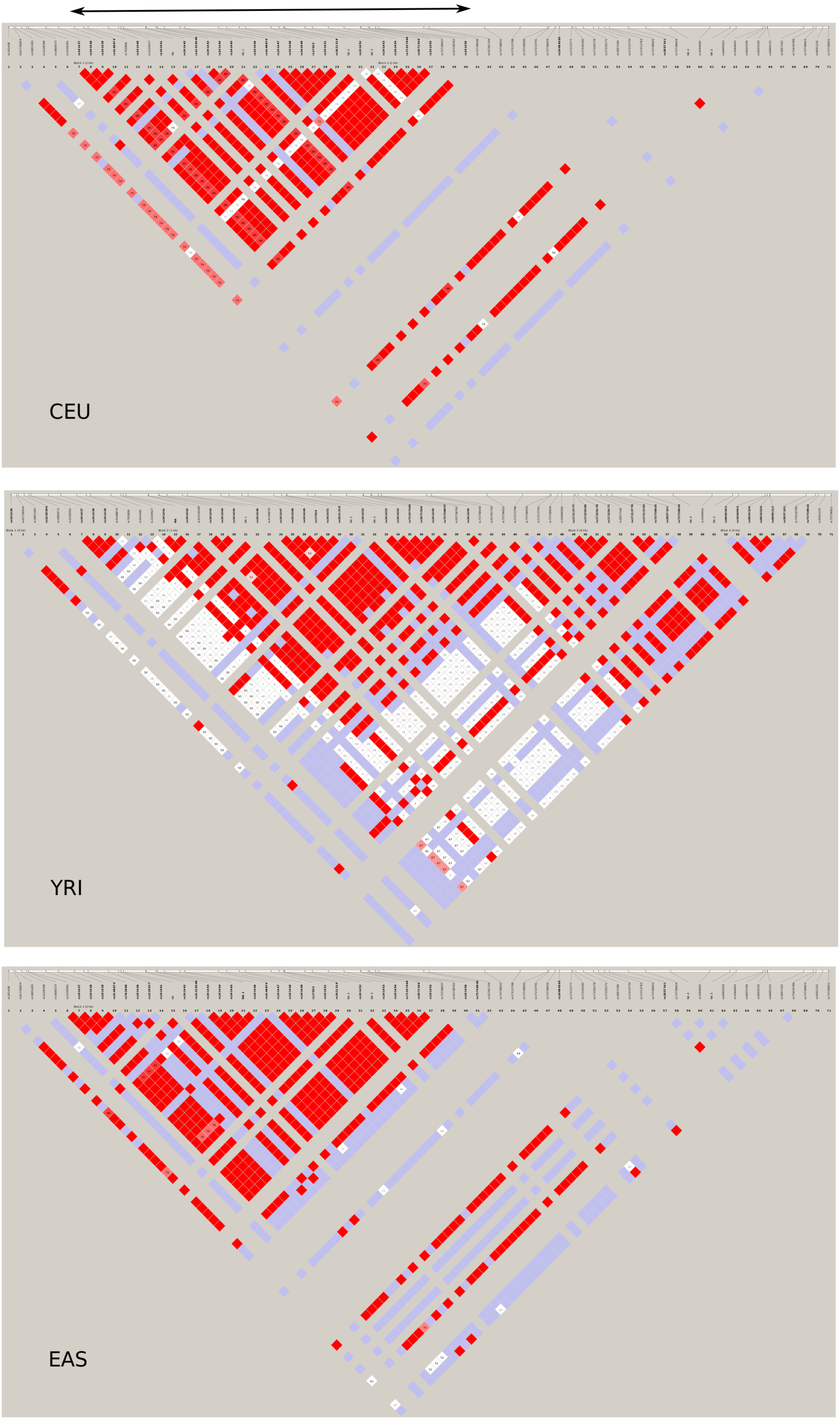

(B)

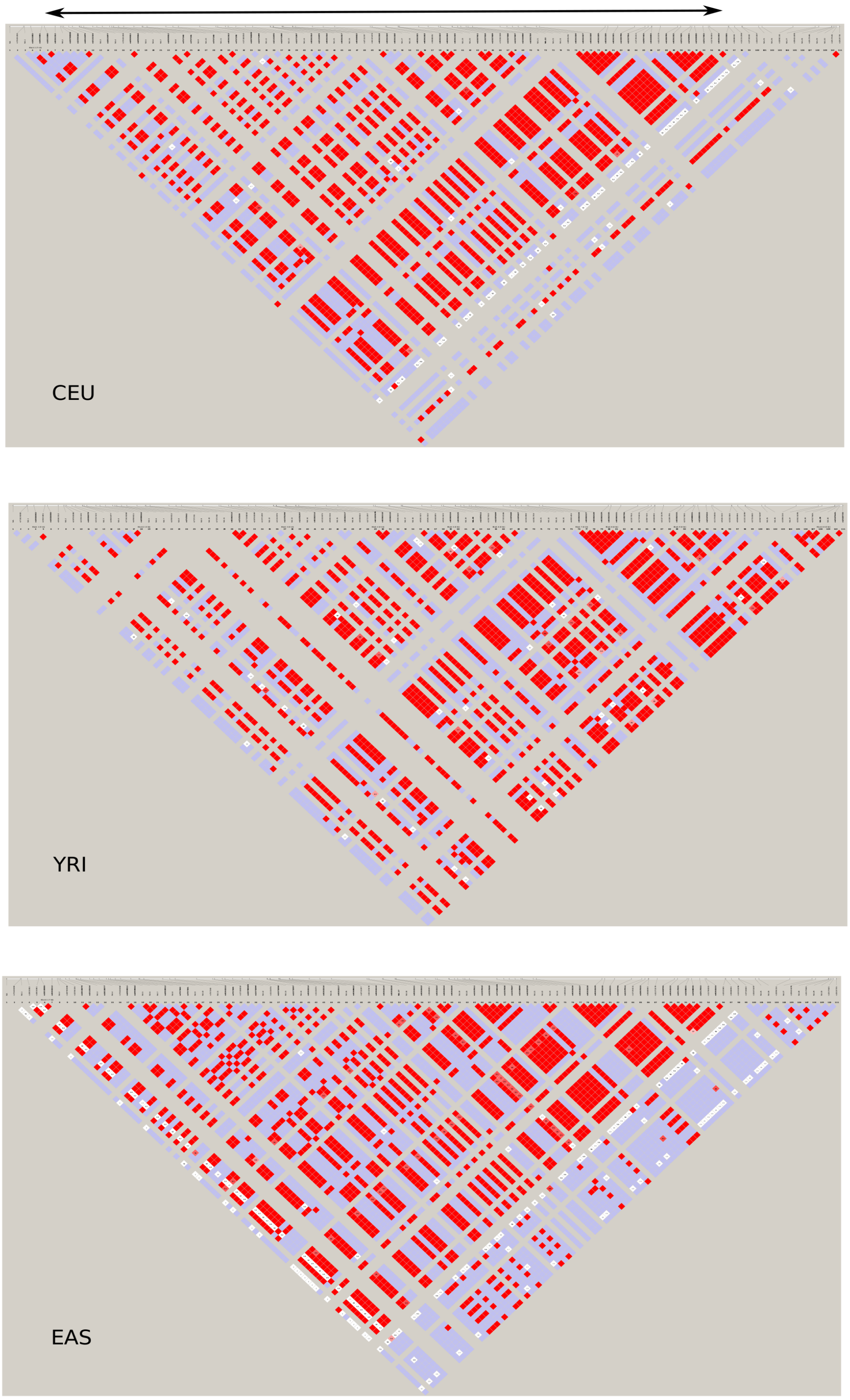

(C)

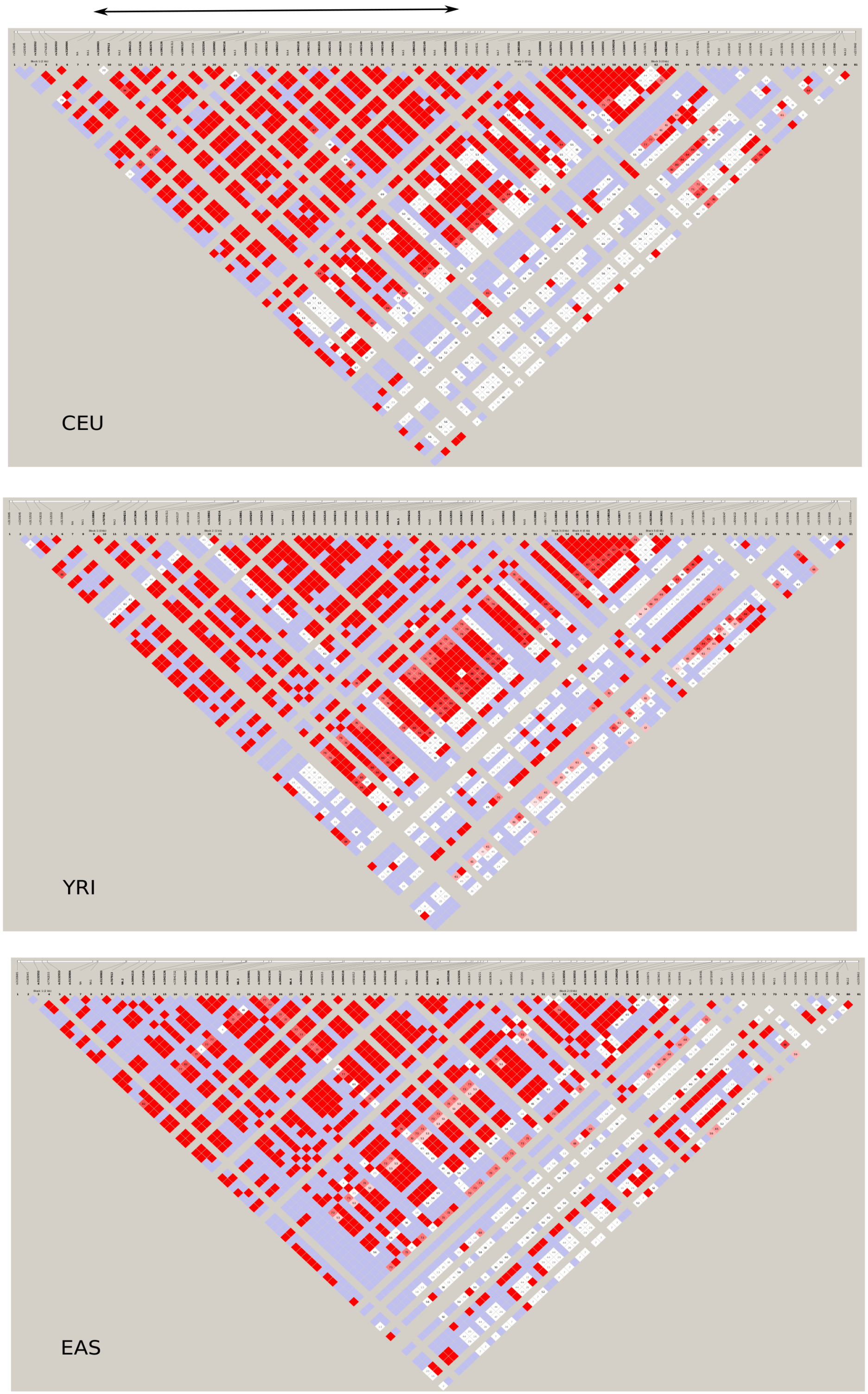

Supplement: Additional file 3 — LD map of the genomic regions we resequenced in TAP2 region (chr6:32901567-32905985), TRIM40/TRIM10 region (chr6:30221583-30230714), CDSN/PSORS1C1 region (chr6:31188857-31193360). [file 1471-2148-11-171-S3.PDF]
