# Supplementary material for: Balancing selection is common in the extended MHC region but most alleles with opposite risk profile for autoimmune diseases are neutrally evolving
Source: BMC Evol Biol. 2011 Jun 17;11:171. doi: 10.1186/1471-2148-11-171 (PMC3141431; doi:10.1186/1471-2148-11-171)
Supplement: Additional File 4 — Nucleotide diversity estimates and summary statistics for the sub-regions included and excluded from GENETREE analysis. [file 1471-2148-11-171-S4.PDF]

Additional File 4. Nucleotide diversity and summary statistics for sub-regions included or excluded from GENETREE analysis.

Gene	L[a]	P[b]	S[c]	θ_W[d]		π[e]		Tajima's D		Fu & Li's D*		Fu & Li's F*	
				value	rank[f]	value	rank[f]	value	rank[f]	value	rank[f]	value	rank[f]
TAP2 (included)	1.9	YRI	22	27.58	>0.99	32.95	>0.99	0.65	0.90	1.37	0.99	1.34	0.99
		CEU	19	23.82	0.99	41.01	>0.99	2.37	0.98	1.65	0.98	2.23	0.98
		EAS	22	27.58	>0.99	46.39	>0.99	2.28	0.99	1.69	>0.99	2.24	0.99
TRIM10 / TRIM40 (included)	7.3	YRI	46	14.83	0.91	13.66	0.92	-0.28	0.57	1.04	0.97	0.69	0.92
		CEU	48	15.48	0.98	24.19	0.99	2.00	0.96	1.57	0.98	2.05	0.98
		EAS	59	19.02	0.99	17.57	0.97	-0.27	0.45	-0.38	0.42	-0.41	0.41
CDSN / PSORS1C1 (included)	2.0	YRI	20	23.37	0.99	35.95	>0.99	1.78	>0.99	1.66	>0.99	2.01	>0.99
		CEU	19	22.20	0.99	40.12	>0.99	2.65	0.98	1.65	0.98	2.34	0.98
		EAS	23	26.88	>0.99	39.88	>0.99	1.62	0.93	1.40	0.97	1.75	0.98
TAP2 (excluded)	2.5	YRI	32	29.79	>0.99	36.00	>0.99	0.72	0.94	1.57	>0.99	1.52	0.99
		CEU	14	13.03	0.95	20.42	0.98	1.79	0.96	1.08	0.88	1.55	0.95
		EAS	19	17.69	0.99	23.09	0.98	1.00	0.79	0.57	0.75	0.84	0.80
TRIM10 / TRIM40 (excluded)	1.8	YRI	18	24.51	0.99	24.76	0.99	-0.0077	0.68	0.57	0.86	0.44	0.82
		CEU	20	26.12	0.99	34.57	>0.99	1.07	0.81	1.32	0.93	1.46	0.94
		EAS	20	26.12	>0.99	26.91	0.99	0.10	0.54	0.28	0.69	0.26	0.63
CDSN / PSORSC1 (excluded)	2.5	YRI	43	40.43	>0.99	52.71	>0.99	1.18	0.98	1.13	0.97	1.36	0.99
		CEU	40	37.62	>0.99	56.95	>0.99	1.81	0.96	1.28	0.93	1.74	0.97
		EAS	41	38.56	>0.99	51.44	>0.99	1.07	0.82	1.13	0.93	1.32	0.92

[a] length of analyzed sequenced region (kb);
[b] population;
[c] number of segregating sites;
[d] Watterson's theta estimation per site (x 10-4);
[e] nucleotide diversity per site (x 10-4);
[f] percentile rank relative to a distribution of 238 5kb segments from NIEHS genes.
