# Supplementary material for: Balancing selection is common in the extended MHC region but most alleles with opposite risk profile for autoimmune diseases are neutrally evolving
Source: BMC Evol Biol. 2011 Jun 17;11:171. doi: 10.1186/1471-2148-11-171 (PMC3141431; doi:10.1186/1471-2148-11-171)
Supplement: Additional file 5 — Table with details on GENETREE analyses. [file 1471-2148-11-171-S5.PDF]

Additional File 5. GENETREE analyses

Region	outgroup	θ_{Ml}	SNPs[a]	N_e	TMRCA (MY)	Standard Deviation (MY)
TAP2	panTro	3.1	8	23250	5.36	0.11
CDSN/PSORS1C1	rheMac	3.5	6	24029	4.18	0.92
TRIM10/TRIM40	panTro	10.5	14	15750	2.49	0.22
BTNL2	ponAbe	9	31	25161	6.04	0.91

[a]Number of SNPs that had to be removed to comply with GENETREE assumptions
